# Lung diffusing capacity for nitric oxide and carbon monoxide following mild‐to‐severe COVID‐19

**DOI:** 10.14814/phy2.14748

**Published:** 2021-02-24

**Authors:** Giovanni Barisione, Vito Brusasco

**Affiliations:** ^1^ Struttura Semplice Fisiopatologia Respiratoria Clinica Malattie Respiratorie e Allergologia Dipartimento di Medicina Interna e Specialità Mediche Università di Genova IRCCS Ospedale Policlinico San Martino Genova Italy; ^2^ Centro Polifunzionale di Scienze Motorie Dipartimento di Medicina Sperimentale Università di Genova IRCCS Ospedale Policlinico San Martino Genova Italy

**Keywords:** alveolar membrane diffusive conductance, carbon monoxide, COVID‐19, ground glass opacities, lung diffusing capacity, nitric oxide

## Abstract

A decreased lung diffusing capacity for carbon monoxide (DL_CO_) has been reported in a variable proportion of subjects over the first 3 months of recovery from severe coronavirus disease 2019 (COVID‐19). In this study, we investigated whether measurement of lung diffusing capacity for nitric oxide (DL_NO_) offers additional insights on the presence and mechanisms of gas transport abnormalities. In 94 subjects, recovering from mild‐to‐severe COVID‐19 pneumonia, we measured DL_NO_ and DL_CO_ between 10 and 266 days after each patient was tested negative for severe acute respiratory syndrome coronavirus 2. In 38 subjects, a chest computed tomography (CT) was available for semiquantitative analysis at six axial levels and automatic quantitative analysis of entire lungs. DL_NO_ was abnormal in 57% of subjects, independent of time of lung function testing and severity of COVID‐19, whereas standard DL_CO_ was reduced in only 20% and mostly within the first 3 months. These differences were not associated with changes of simultaneous DL_NO_/DL_CO_ ratio, while DL_CO_/V_A_ and DL_NO_/V_A_ were within normal range or slightly decreased. DL_CO_ but not DL_NO_ positively correlated with recovery time and DL_CO_ was within the normal range in about 90% of cases after 3 months, while DL_NO_ was reduced in more than half of subjects. Both DL_NO_ and DL_CO_ inversely correlated with persisting CT ground glass opacities and mean lung attenuation, but these were more frequently associated with DL_NO_ than DL_CO_ decrease. These data show that an impairment of DL_NO_ exceeding standard DL_CO_ may be present during the recovery from COVID‐19, possibly due to loss of alveolar units with alveolar membrane damage, but relatively preserved capillary volume. Alterations of gas transport may be present even in subjects who had mild COVID‐19 pneumonia and no or minimal persisting CT abnormalities.

**Trial registry:**

ClinicalTrials.gov PRS: No.: NCT04610554 Unique Protocol ID: SARS‐CoV‐2_DLNO 2020.

## Graphical abstract

An impairment of lung diffusing capacity for nitric oxide exceeding standard DL_CO_ may be present during the recovery from COVID‐19, possibly due to loss of alveolar units with alveolar membrane damage

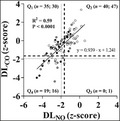

## 1 INTRODUCTION

Infection with the severe acute respiratory syndrome coronavirus 2 (SARS‐CoV‐2) has been the cause, in a variable number of subjects, of a disease named severe coronavirus disease 2019 (COVID‐19) showing clinical manifestations ranging from mild upper airway symptoms to interstitial pneumonia with or without acute hypoxemic respiratory failure (Guan et al., 2020). Among the distinctive features of COVID‐19, in comparison with influenza virus pneumonia, are an increase of serum D‐dimer and, at autopsy, the presence of alveolar damage with widespread thrombotic microangiopathy (Ackermann et al., 2020). SARS‐CoV‐2 targets preferentially type II alveolar cells (Mason et al., 2020), which are the precursors for type I cells; thus, it can be hypothesized that COVID‐19 survivors might develop gas exchange abnormalities because of aberrant alveolar wound healing, or loss of pulmonary vascular bed, or both.

Three preliminary studies found a mild decrement of lung diffusing capacity for carbon monoxide (DL_CO_) in about half of subjects 1 month after symptom onset (Frija‐Masson et al., 2020; Mo et al., 2020) or hospital discharge (Huang et al., 2020). Two studies found DL_CO_ be reduced in 21% (Sonnweber et al., 2020) and 24% (Lerum et al., 2020) of subjects about 3 months after hospital admission, and one study in 34% of subjects 3 months after recovery from the acute phase of disease (van den Borst et al., 2020). Two of these studies (Lerum et al., 2020; Mo et al., 2020) also reported values of DL_CO_‐to‐alveolar volume (DL_CO_/V_A_) ratio, that is, K_CO_, to be slightly decreased (Mo et al., 2020) or within the normal range (Lerum et al., 2020) in the majority of subjects, which would suggest an alveolar damage associated with diffuse microvascular destruction (Hughes & Pride, 2012). However, the interpretation of the above findings is complicated by differences in the cutoffs for defining DL_CO_ abnormality, coexisting comorbidities, time of lung function studies, and severity of disease in the acute phase. Moreover, the major limit to lung CO uptake is its slow binding with intracapillary hemoglobin (Hb), which makes DL_CO_ unable to distinguish between reductions of alveolar membrane diffusive conductance (DM) and pulmonary capillary blood volume (V_C_) (Borland & Hughes, 2020; Guénard et al., 1987). By contrast, nitric oxide (NO) has a much greater affinity and faster reaction rate with Hb than CO (Gibson & Roughton, 1957), which make the lung diffusing capacity for NO (DL_NO_) more sensitive to changes in DM than V_C_ (Borland & Hughes, 2020; Guénard et al., 1987). Indeed, recent studies on interstitial lung diseases (Barisione et al., 2016, 2019) have shown that DL_NO_ reflects fibrotic changes more accurately than standard DL_CO_.

Thus, considering the complex pathophysiology of COVID‐19 (Ackermann et al., 2020; Mason et al., 2020), we undertook the present study to investigate whether measurements of DL_NO_ and DL_CO_ can provide different information on gas exchange abnormalities persisting after COVID‐19 that may be related to radiological findings, severity of pneumonia, and time of recovery.

## 2 MATERIALS AND METHODS

### 2.1 Study subjects

This study included 94 Caucasian subjects who attended our pulmonary function laboratory as outpatients for follow‐up after in‐hospital treatment for COVID‐19 pneumonia, confirmed by ground glass opacities (GGO) or band‐like consolidations on chest roentgenogram or computed tomography (CT) and positive nasopharyngeal swabs for SARS‐CoV‐2. Pulmonary function tests were obtained between 10 and 266 days after hospital discharge, which occurred only after each patient had been tested negative for SARS‐CoV‐2. To be included in the study, subjects were required not to have history of comorbidities potentially affecting lung diffusing capacity, that is, bronchial asthma, chronic obstructive pulmonary disease, pulmonary interstitial fibrosis or vasculitis, systemic collagen disease, congestive heart failure, liver or renal diseases, and morbid obesity. They were classified in three groups based on the presence or severity of acute hypoxemic respiratory failure and the respiratory support received during hospitalization (Table 1). Acute hypoxemic respiratory failure was diagnosed whenever the measured oxygen partial pressure (PaO_2_) in an arterial blood sample drawn from the radial artery during room air breathing was below the age‐adjusted lower limit of normal (Cerveri et al., 1995). The first group included 34 subjects who had no arterial hypoxemia, a second group included 34 subjects who had mild‐to‐moderate arterial hypoxemia treated by O_2_‐supplementation with (*n *=* *31) or without (*n *=* *3) helmet continuous positive airway pressure, and a third group included 26 subjects who had severe arterial hypoxemia treated by O_2_‐supplementation and invasive mechanical ventilation via tracheal intubation (*n *=* *23) or tracheostomy (*n *=* *3). During hospitalization, they had received antibiotics (*n *=* *63), oral hydroxychloroquine (*n *=* *49), corticosteroids (*n *=* *43), enoxaparin (*n *=* *37), tocilizumab or anakinra,

**TABLE 1:** Subjects’ anthropometric characteristics and lung function data (*n *=* *125)

	Controls	COVID‐19 Severity			*p* value
		Mild	Moderate	Severe	
Male/Female	22/9	21/13	21/13	23/3	0.20
Age (years)	57 ± 12	62 ± 14	61 ± 10	60 ± 11	0.38
Stature (cm)	171 ± 11	167 ± 9	169 ± 10	171 ± 8	0.29
BMI (kg·m^−2^)	25 ± 3	27 ± 4	29 ± 4*	28 ± 4	0.003
Smokers (current‐former/never)	16/15	22/12	18/16	12/14	0.41
FVC (L)	4.68 ± 1.28	4.00 ± 0.88*	3.90 ± 1.04*	4.00 ± 0.87	0.011
(% predicted)	112 ± 14	108 ± 14	102 ± 16*	97 ± 15* ^,^ ^†^	<0.001
(*z*‐score)	0.79 ± 0.93	0.53 ± 0.89	0.11 ± 1.11*	−0.25 ± 0.98* ^,^ ^†^	<0.001
FEV_1_ (L)	3.61 ± 0.99	3.04 ± 0.71*	3.08 ± 0.77*	3.10 ± 0.66	0.015
(% predicted)	110 ± 13	105 ± 14	104 ± 18	96 ± 15*	0.004
(*z*‐score)	0.71 ± 0.90	0.37 ± 0.96	0.25 ± 1.17	−0.25 ± 0.99*	0.003
TLC (L)	6.68 ± 1.43	5.86 ± 1.01*	5.60 ± 1.24*	5.68 ± 1.28*	0.003
(% predicted)	106 ± 12	100 ± 13	94 ± 15*	87 ± 14* ^,^ ^†^	<0.001
(*z*‐score)	0.51 ± 1.08	−0.06 ± 1.10	−0.61 ± 1.42*	−1.16 ± 1.33* ^,^ ^†^	<0.001
DL_CO_ (mL·min^−1^·mmHg^−1^)	30.3 ± 8.80	23.2 ± 6.71*	21.4 ± 6.65*	22.4 ± 5.60*	<0.001
(% predicted)	118 ± 19	100 ± 22*	89 ± 20*, †	87 ± 19* ^,^ ^†^	<0.001
(*z*‐score)	0.99 ± 1.04	−0.06 ± 1.29*	−0.80 ± 1.41*, †	−0.91 ± 1.30* ^,^ ^†^	<0.001
DL_CO_/V_A_ (mL·min^−1^·mmHg^−1^·L^−1^)	4.52 ± 0.69	4.04 ± 0.77	3.90 ± 0.76*	4.04 ± 0.74*	0.005
(% predicted)	103 ± 15	94 ± 17	90 ± 19*	92 ± 16*	0.007
(*z*‐score)	0.22 ± 0.93	−0.41 ± 1.08	−0.68 ± 1.26*	−0.59 ± 1.04*	0.007
DL_NO_ (mL·min^−1^·mmHg^−1^)	124.8 ± 37.1	96.9 ± 29.7*	89.5 ± 28.5*	91.7 ± 23.0*	<0.001
(% predicted)	90 ± 10	78 ± 17*	69 ± 15*	65 ± 13* ^,^ ^†^	<0.001
(*z*‐score)	−0.69 ± 0.71	−1.44 ± 1.10*	−1.98 ± 1.05*	−2.47 ± 1.02* ^,^ ^†^	<0.001
DL_NO_/V_A_ (mL·min^−1^·mmHg^−1^·L^−1^)	19.2 ± 3.02	17.8 ± 3.40	17.3 ± 3.14	17.3 ± 3.06	0.06
(% predicted)	88 ± 9	86 ± 14	82 ± 13	82 ± 13	0.10
(*z*‐score)	−0.90 ± 0.70	−1.08 ± 1.02	−1.38 ± 0.96	−1.40 ± 0.99	0.10
DL_NO_ /DL_CO_	4.22 ± 0.43	4.19 ± 0.59	4.34 ± 0.66	4.35 ± 0.43	0.37

Data are absolute numbers or mean ± SD; FVC, forced vital capacity; FEV_1_, forced expiratory volume in 1 second; TLC, total lung capacity; DL_CO_, standard single‐breath lung diffusing capacity for carbon monoxide; DL_NO_, single‐breath lung diffusing capacity for nitric oxide; V_A_, alveolar volume; DL_NO_/DL_CO_, ratio of simultaneous DL_NO_ and DL_CO_ measurements.

* Significantly different from controls.

† Significantly different from mild group.

(*n *=* *24), and various antiviral drugs (*n *=* *18). As a control group, we selected 31 healthy subjects, matched for anthropometric characteristics and smoking habit, among health professionals and their relatives studied before the onset of COVID‐19 pandemic.

### 2.2 Lung function measurements

Spirometry (Graham et al., 2019) and lung volumes (Wanger et al., 2005) were determined with subjects sitting in a whole‐body plethysmograph (V62 J, SensorMedics‐Viasys, CareFusion; Höchberg, Germany) and breathing quietly with a nose clip in place. Forced vital capacity (FVC), forced expiratory volume in one second (FEV_1_), their ratio (FEV_1_/FVC), and total lung capacity (TLC) were measured and compared with predicted values (Quanjer et al., 1993, 2012).

Standard DL_CO_ was measured (MasterScreen PFT System, Jaeger‐Viasys, CareFusion, Höchberg, Germany) by single‐breath technique with a measured breath‐hold time of 11 ± 0.4 s. Maneuvers with inspired volume ≥85% of vital capacity, 8–12 s breath‐hold time, and sample collection ≤4 s were retained for analysis (Graham et al., 2017). Results were compared with the predicted values from Stanojevic et al. (2017) after adjustment for effective Hb measured in available arterial or venous blood samples ($\left[{Hb}_{meas}\right]$ (Cotes et al., 1972).

At least 5–10 min after standard DL_CO_, single‐breath DL_NO_ and DL_CO_ were simultaneously measured with an actual breath‐hold time of 5 ± 0.3 s as detailed elsewhere (Barisione et al., 2016, 2019), and the DL_NO_/DL_CO_ ratio calculated. Predicted values for DL_NO_ and DL_NO_/V_A_ were from Zavorsky et al. (2017).

Personnel wearing equipment against exposure to SARS‐CoV‐2 did all testing and instrument cleaning disinfection procedures.

### 2.3 Chest CT

In 38 subjects, a thin‐section CT scan obtained between 0 and 207 days after hospital discharge and 34 days (median 8 days; interquartile range 25–75% [IQR_25‐75%_] 0–19) before or after pulmonary function measurements was available. Scans of the entire chest were obtained in a supine position, during breath‐holding at full inspiration, by a multi‐detector row‐spiral scanner (SOMATOM Emotion 6, Siemens AG Medical, Forchheim, Germany). Images were acquired by 110 kVp tube voltage at 1.25‐mm slice thickness and reconstructed at 1‐mm increments using smooth (B41 s) and sharp (B70 s) convolution kernels. CT scans acquired at an absolute lung volume ≥80% of plethysmographic TLC were retained for semiquantitative calculation of voxel percentages with GGO at six axial levels (Barisione et al., 2016, 2019) and automatic quantitative 3D analysis of mean lung attenuation (MLA) and its coefficient of variation (MLA CV %) for the entire lung (ITK‐Snap 3.8.0, Philadelphia, PA, US) (Yushkevich et al., 2006).

### 2.4 Statistical analysis

For each lung function measure, we calculated the percentage of predicted and *z*‐score values. As lower limits of normality for DL_NO_ and standard DL_CO_, we considered both 5th (LLN_5_, *z*‐score −1.645) and 2.5th (LLN_2.5_, *z*‐score −1.96) percentiles of the reference population. Categorical variables were compared by z‐test with Yates correction, while Fisher's exact test was used to compare their distributions. Continuous variables were tested by one‐way pairwise ANOVA with Holm‐Sidak post hoc test for multiple comparisons. Associations between variables were tested for significance by the coefficient of

determination (R^2^). The difference between two dependent correlations with one variable in common was calculated by an asymptotic two‐tailed *z*‐test, with values >1.96 considered significant (Steiger 1980). Data are presented as mean ±SD or median with IQR_25‐75%_ whenever appropriate. In all analyses, the acceptable type I error was set at *p* <* *0.05.

## 3 RESULTS

Collectively, all standard lung function measures and DL_NO_ were significantly lower in the three COVID‐19 groups than in the control group, whereas DL_NO_/V_A_ and DL_NO_/DL_CO_ ratios did not differ significantly.

**FIGURE 1:**
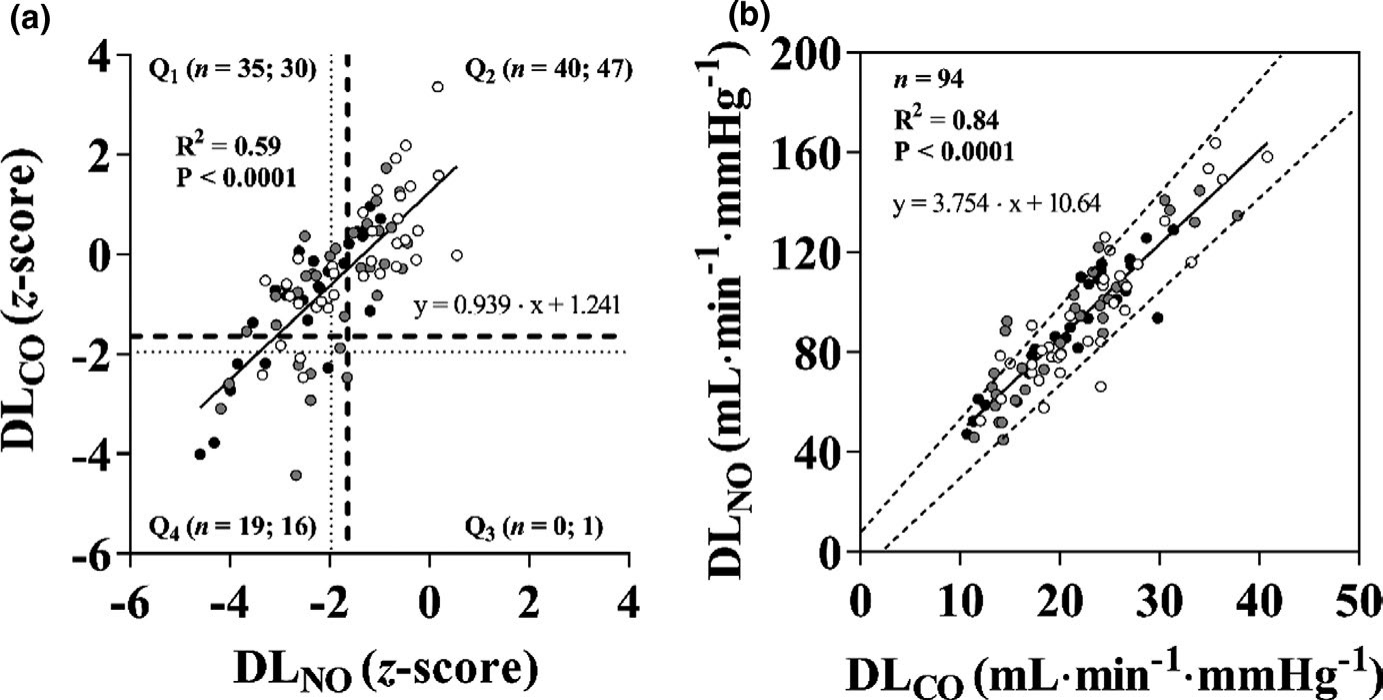
Panel a: Relationship between *z*‐scores of standard lung diffusing capacity for carbon monoxide (DL_CO_) and lung diffusing capacity for nitric oxide (DL_NO_). Horizontal and vertical lines correspond to the 5th (dashed) and 2.5th (dotted) percentiles of reference values, that is, −1.645 and −1.96 *z*‐scores, respectively. The numbers within brackets indicate the subjects falling into each quadrant (Q_1_‐Q_4_) bounded within 5th or 2.5th percentiles. Symbols indicate subjects recovering from *mild* (white), *moderate* (gray), and *severe* (black) COVID‐19 pneumonia. Panel b: Correlation between simultaneous measures of DL_NO_ and DL_CO_. Upper and lower oblique dashed lines indicate the 95% confidence interval for DL_NO_/DL_CO_ ratio in healthy controls

There was a significant correlation between DL_NO_ and standard DL_CO_ *z*‐scores (R^2^: 0.59; *p* < 0.0001) (Figure 1a). However, considering individual data, 35 subjects (37%) had DL_NO_ but not DL_CO_ below the LLN_5_, and 30 of them also below the LLN_2.5_, 19 subjects (20%) had both DL_NO_ and DL_CO_ below the LLN_5_ and 16 of them also below the LLN_2.5_, 40 subjects (43%) had both DL_NO_ and DL_CO_ above the LLN_5_ and 47 of them also above the LLN_2.5_, and only one subject had DL_CO_ but not DL_NO_ below the LLN_2.5_. There were no significant differences in the distribution of subjects with reduced DL_NO_, DL_CO_, or both in relation to the presence or severity of acute hypoxemic respiratory failure and type of respiratory support received during hospitalization. The DL_NO_/DL_CO_ ratio was in the majority of COVID‐19 subjects within 1.96 SD of the values observed in the control group (Figure 1b).

**FIGURE 2:**
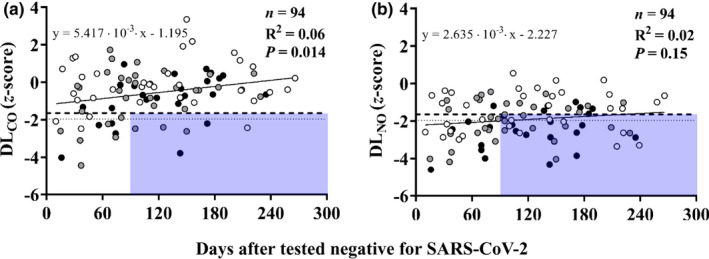
Relationships between standard DL_CO_ (panel a) or DL_NO_ (panel b) and time elapsed from negative testing for SARS‐CoV‐2 to lung function studies. Symbols indicate subjects who recovered from *mild* (white), *moderate* (gray), and *severe* (black) COVID‐19 pneumonia. Horizontal lines correspond to the 5th (dashed) and 2.5th (dotted) percentiles of reference values, that is, −1.645 and −1.96 *z*‐scores, respectively. The shaded areas include the subjects with abnormal standard DL_CO_ or DL_NO_ values after the first 3 months of recovery

There was a weak albeit significant positive correlation between standard DL_CO_ (R^2^ = 0.06; *p* = 0.014) but not DL_NO_ (R^2^ = 0.02; *p* = 0.15) and the time elapsed between negative test for SARS‐CoV‐2 and lung function studies (Figure 2a,b). Notably, of the 58 subjects studied after 3 months, 30 had DL_NO_ below the LLN_5_ and 25 also below the LLN_2.5_, while only six had DL_CO_ below the LLN_5_ and LLN_2.5_ (*p* < 0.001).

**FIGURE 3:**
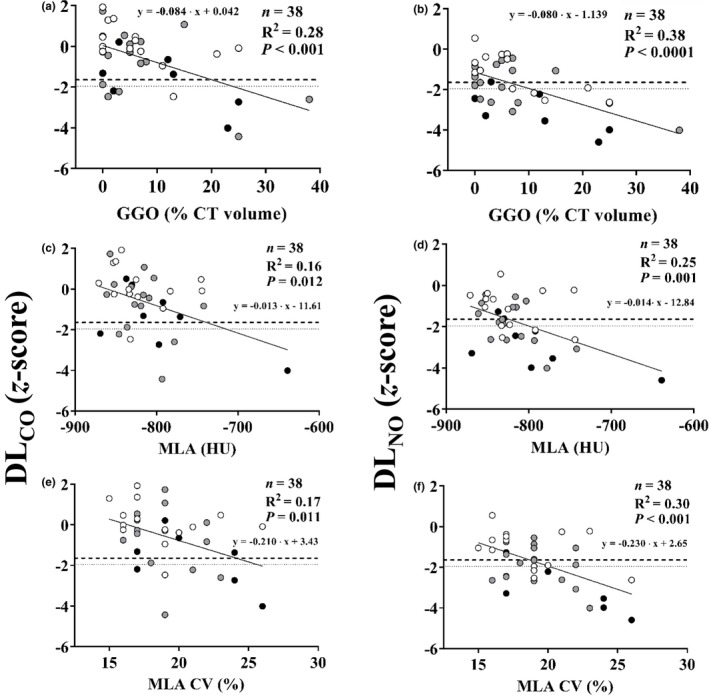
Correlations between standard DL_CO_ (panels a, c, and e), or DL_NO_ (panels b, d, and f) and ground glass opacities (GGO), as percentage of total CT volume, mean lung attenuation (MLA) in Hounsfield units (HU), and its coefficient of variation (MLA CV%). Symbols indicate subjects who recovered from *mild* (white), *moderate* (gray), and *severe* (black) COVID‐19 pneumonia. Horizontal lines correspond to the 5th (dashed) and 2.5th (dotted) percentiles of reference values, that is, −1.645 and −1.96 *z*‐scores, respectively

**FIGURE 4:**
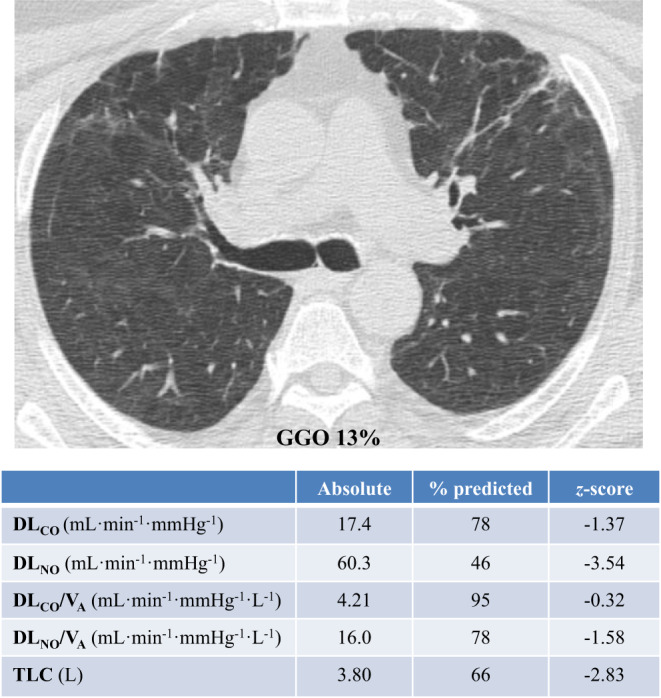
Axial CT scan acquired at the bifurcation of main bronchi (carina) in supine position in a representative subject who had severe COVID‐19 pneumonia treated by invasive mechanical ventilation. Note the discrepancy between DL_NO_ and standard DL_CO_ in the presence of moderate GGO extent. Abbreviations as in Table 1

The CT scans obtained within 34 days from lung function studies showed GGO above 5% of total lung volume be present in 21 (55%) of the 38 subjects examined (Figure 3a,b). Both DL_NO_ and standard DL_CO_ *z*‐scores were inversely related to the extent of GGO with correlation coefficients insignificantly different (*p* = 0.61) between each other but *y*‐intercepts significantly (*p* < 0.0001) lower for DL_NO_ than standard DL_CO_. Therefore, reduced DL_NO_ was associated with GGO more frequently than DL_CO_. Similar correlations were observed between DL_NO_ or standard DL_CO_ with MLA or MLA CV% (Figure 3c‐f). Figure 4 shows an example of wide discrepancy between DL_NO_ and standard DL_CO_ in a subject with moderate CT abnormality. Quantitative analysis of the entire lung and qualitative analysis at six axial levels did not reveal areas of reticular opacities, honeycombing, or hypoattenuation (<−950 HU) in any subject.

## 4 DISCUSSION

The main findings of the present study are that *1)* abnormal DL_NO_ was present in more than half of the subjects over 8 months of recovery from mild‐to‐severe COVID‐19 pneumonia, whereas standard DL_CO_ was abnormal in only 20%, *2)* standard DL_CO_ but not DL_NO_ was positively correlated with recovery time, and *3)* both standard DL_CO_ and DL_NO_ were inversely correlated with persisting CT abnormalities, but DL_NO_ was more frequently associated with their presence.

### 4.1 Comments on methodology

**FIGURE 5:**
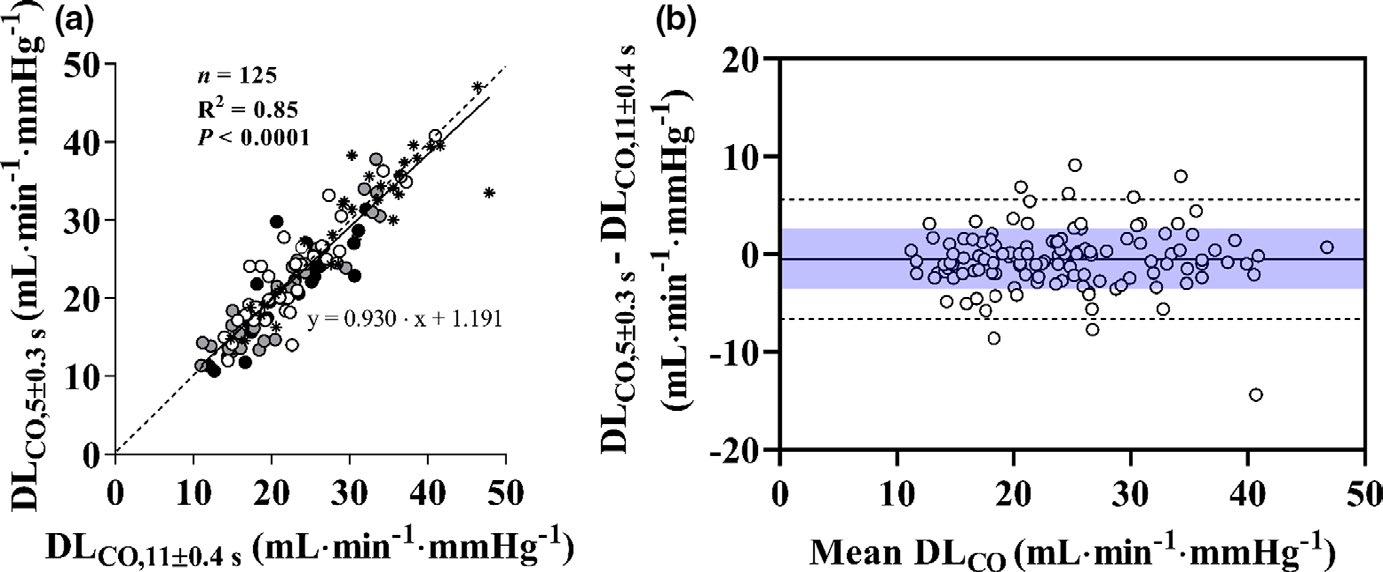
Panel a: Correlation between absolute values of DL_CO_ measured by standard method with breath‐hold time of 11 ± 0.4 s (DL_CO,11±0.4 s_) or by simultaneous DL_NO_‐DL_CO_ method with breath‐hold time of 5 ± 0.3 s (DL_CO,5±0.3 s_). Asterisks (*) indicate healthy controls while circles indicate subjects who recovered from *mild* (white), *moderate* (gray), and *severe* (black) COVID‐19 pneumonia. Panel b: Bland‐Altman plot of difference vs. mean DL_CO_ measured by the two methods. Shaded area is the standard deviation of differences, and horizontal dashed lines indicate the 95% confidence interval

In this study, we measured DL_CO_ by standard technique and in combination with DL_NO_, which required breath‐hold times of 11 ± 0.4 s and 5 ± 0.3 s, respectively. Such a difference seems to have a negligible effect on final values of DL_CO_ both in healthy subjects and restrictive disorders, that is, idiopathic pulmonary fibrosis (Barisione et al., 2016) and systemic sclerosis‐associated interstitial lung disease (Barisione et al., 2019). Also in the present investigation, absolute values of DL_CO_ measured by the two methods were strongly correlated (R^2^ = 0.85; *p* < 0.0001) (Figure 5a) without systematic differences (Figure 5b). Therefore, we used standard DL_CO_ values for comparison with DL_NO_ and the results of previous studies.

Although the 5th percentile (*z*‐score −1.645) is generally assumed as the lower limit of normal for standard lung function measurements including DL_CO_ (Quanjer et al., 1993), the 2.5th percentile (*z*‐score −1.96) has been suggested for DL_NO_ with the currently available predictive equations (Munkholm et al., 2018; Zavorsky et al., 2017). Therefore, we have used both LLN_5_ and LLN_2.5_ to reduce false negative or false positive biases. As reference values for DL_NO_ and DL_NO_/V_A_, we used the set of equations that provided the lower SD of *z*‐scores from our local data set of healthy subjects, that is, 0.71 and 0.70, respectively.

The alveolar concentration of endogenous NO increases in several inflammatory interstitial lung diseases (Cameli et al., 2020), which could theoretically bias DL_NO_ measures. However, the mean NO concentration in the gas mixtures inhaled in the present study was 63.7 ± 10 ppm, resulting in alveolar concentrations ranging from 5.4 to 21.9 ppm, thus >1,000 times the threshold considered as a marker of pulmonary alveolitis. Hence, it is reasonable to assume that any effect of endogenous NO backpressure on DL_NO_ measurements was negligible. Furthermore, 40 ppm of NO in the inspired gas could decrease hypoxic pulmonary vasoconstriction (Glenny & Robertson, 2011), but this effect was observed with$P{AO}_{2}$<60 mmHg (Asadi et al., 2015), thus well below the 102 ± 4 mmHg of this study.

The present study has two major limitations. *First*, lung function tests were obtained in a sitting posture and CT in supine position. The latter might have increased V_C_ (Cotton et al., 1990), thus possibly affecting differently the relationships of DL_NO_ and standard DL_CO_ with CT density distribution data. *Second*, the study was cross‐sectional, which may limit the clinical relevance of results but does not seem to invalidate their pathophysiological meaning and interpretation.

### 4.2 Comments on results

To our knowledge, this is the first study using DL_NO_ and DL_CO_ to investigate the pathophysiology of alveolar‐to‐capillary gas exchange in patients recovering from COVID‐19. Clinically, COVID‐19 pneumonia is associated in a variable number of subjects with acute hypoxemic respiratory failure ranging from mild‐to‐severe, whereas other subjects have no apparent gas exchange abnormalities (Guan et al., 2020). At autopsy of patients who died from severe COVID‐19, diffuse alveolar damage, capillary endothelialitis, and fibrinous microthrombi with angiogenesis within the interalveolar septa has been observed (Ackermann et al., 2020). A question is whether these abnormalities occurring in the acute phase of the disease might leave late pathophysiological sequelae over the recovering phase and these depend on the presence or severity of acute hypoxemic respiratory failure. A mild reduction of standard DL_CO_ has been reported in about half of survivors as early as 30 days after acute infection (Frija‐Masson et al., 2020; Mo et al., 2020) or hospital discharge (Huang et al., 2020). In the present study, we found a much lower prevalence of decreased standard DL_CO_, that is, 20% and 18% with LLN_5_ and LLN_2.5_, respectively, over 8 months after negative SARS‐CoV‐2 testing. There are three main reasons that may have contributed to this discrepancy. *First*, we used lower limits of normal based on *z*‐scores instead of 80% of predicted (Huang et al., 2020; Mo et al., 2020), which tends to overestimate the presence of abnormality due to age‐, sex‐, and size biases (Miller & Brusasco, 2016). Indeed, our results are in keeping with the decrease of DL_CO_ found in 24% of subjects in one study using *z*‐scores (Lerum et al., 2020). *Second*, the proportion of subjects with reduced DL_CO_ tended to decrease with the time elapsed from the negative testing for SARS‐CoV‐2 as suggested by Sonnweber et al. (2020). Instead, we found that more than half of subjects had DL_NO_ below the LLN_5_ and 49% below the LLN_2.5_, and this proportion remained near constant over 8 months. *Third*, almost all previous studies included several patients with comorbidities potentially affecting the final value of DL_CO_ independent of COVID‐19 severity (van den Borst et al., 2020; Frija‐Masson et al., 2020; Huang et al., 2020; Mo et al., 2020; Sonnweber et al., 2020). Collectively, our results support the hypothesis that a more severe and prolonged abnormality of DL_NO_ may be present after COVID‐19 pneumonia, reflecting a prevailing decrement of DM.

Several physiological mechanisms can explain a disproportionate reduction of DL_NO_ and DL_CO_. Since the alveolar‐to‐capillary transfer of CO is mostly limited by its slow reaction rate with Hb (Carlsen & Comroe, 1958), DL_CO_ is relatively less sensitive to changes in DM than V_C_. By contrast, NO has a much greater affinity and fast reaction rate with Hb, which make DL_NO_ more sensitive to DM than V_C_ (Borland & Hughes, 2020). Thus, the findings of the present study suggest that a decreased DM is more frequent and persistent than the reduction of V_C_ in the recovery phase after COVID‐19 pneumonia. One reason for decreased DM could be simply a loss of lung volume, but this would have caused an increase of DL_NO_/V_A_, which was instead slightly below the LLN_5_ or LLN_2.5_ in about one third of subjects. Moreover, TLC was significantly lower than in controls and in subjects with moderate‐to‐severe than mild pneumonia, while there were no differences in the distribution of DL_NO_ and DL_CO_ abnormalities. DL_NO_/DL_CO_ ratio was in most cases within the normal range suggesting that alveolar damage rather than loss of lung volume was the major determinant of diffusion limitation (Hughes & van der Lee, 2013). A possible mechanism for the differences in DL_NO_ and DL_CO_ over recovery time could be that SARS‐CoV‐2, by targeting type II and eventually type I pneumocytes (Mossel et al., 2008), may cause a persistent damage of alveolar membrane while vasculopathy with capillary microthrombi is possibly reversing more rapidly after the acute phase of the disease. However, while V_C_ reflects pulmonary blood volume only, DM reflects alveolar membrane thickness and surface but also vessel surface (Kang & Sapoval, 2016). The latter may be reduced as a consequence of capillary remodeling or obliteration with blood volume being redistributed to unaffected lung regions (Oppenheimer et al., 2006; Pande et al., 1975), or uneven red cell distribution within the alveolar capillaries (Hsia et al., 1997). Another reason for decreased DM without V_C_ changes could be the presence of interstitial edema (Zavorsky et al., 2014), which would be consistent with the closer associations of DL_NO_ than standard DL_CO_ with CT abnormalities.

In the present study, GGO was the only qualitative CT abnormality persisting after COVID‐19 and correlated with decrement of DL_NO_ and standard DL_CO_. In interstitial pulmonary fibrosis (Barisione et al., 2016) or interstitial lung disease associated with systemic sclerosis (Barisione et al., 2019), we found DL_NO_ be correlated with CT fibrotic abnormalities but not GGO. This may suggest that interstitial edema by itself may not be sufficient to alter substantially the alveolar‐to‐capillary gas transport, owing to the high solubility of both NO and CO (Wilhelm et al., 1977). Moreover, we observed reduced DL_NO_ even in the absence or minimal‐to‐moderate GGO, which suggests that mechanisms other than alveolar membrane thickening may contribute to diffusion abnormality after COVID‐19.

## 5 CONCLUSIONS

In subjects recovering from COVID‐19 pneumonia, DL_NO_ is impaired more frequently and more persistently than standard DL_CO_, suggesting an impairment of DM due to alveolar‐capillary damage and loss of alveolar units with V_C_ relatively preserved. DL_NO_ was more frequently abnormal than standard DL_CO_ even in subjects with minimal or absent CT abnormalities, suggesting persistent alveolar damage in these subjects. Further long‐term studies are necessary to investigate whether these medium‐term changes may evolve into chronic morphological and functional abnormalities.

## CONFLICT OF INTEREST

G.B. and V.B have no financial/nonfinancial interests to disclose.

## AUTHOR CONTRIBUTIONS

G.B. conceived and designed the research and performed the experiments; G.B. and V.B. analyzed the data; G.B. and V.B. interpreted results of experiments; G.B. prepared figures; G.B. and V.B. drafted the manuscript; G.B. and V.B. edited and revised the manuscript; and G.B. and V.B. approved the final version of the manuscript.

## ETHICAL APPROVAL

The study was approved by the Regional Ethics Committee (CER Liguria Registry No.: 412/2020 ‐ DB id 10794) and each subject gave written informed consent to use his/her anonymized personal data.
